# Awareness of Child Abuse and Neglect: A Prospective Interventional Study among Schoolteachers from Andhra Pradesh

**DOI:** 10.3390/pediatric16010015

**Published:** 2024-02-27

**Authors:** Anuja Singaraju, Venkata Ratna Kumar Rudravaram, Sivakumar Nuvvula, Sreekanth Kumar Mallineni

**Affiliations:** 1 Department of Paediatric and Preventive Dentistry, Narayana Dental College and Hospital, Nellore 524003, Andhra Pradesh, India; 2 Rewards Dental, Seattle, WA 14212, USA; 3 Pediatric Dentistry, Dr. Sulaiman Al Habib Hospital, Ar Rayyan, Riyadh 14212, Saudi Arabia; 4 Division for Globalization Initiative, Liaison Center for Innovative Dentistry Graduate School of Dentistry, Tohoku University, Sendai 980-8575, Japan

**Keywords:** child abuse, neglect, maltreatment, teachers, audiovisual aid, India

## Abstract

Aim: To assess the awareness of schoolteachers from Andhra Pradesh towards child abuse and neglect (CAN) through pre- and post-educational intervention (audiovisual aid) questionnaires. Materials and methods: A cross-sectional study was conducted with 300 schoolteachers using a 12-item questionnaire that was created using the standard focus group discussion method. Baseline awareness of CAN was assessed using the questionnaire. Subsequently, all the schoolteachers were educated regarding the various types of CAN and the process of reporting CAN using an audiovisual aid. The same questionnaire was re-administered to all the teachers immediately after the intervention and after three months. The data were statistically analyzed using Fisher’s exact test to compare the frequency and distribution of responses among the study participants at various intervals. Results: A statistically significant difference (*p* value < 0.05) was observed in the awareness of the schoolteachers regarding CAN compared to the baseline and immediately after the intervention. However, there was no statistically significant difference (*p* value > 0.05) between immediately after the intervention and three months. Conclusion: There is a need to have awareness among schoolteachers concerning CAN. However, after education through audiovisual aids, teachers’ awareness of CAN has been improved.

## 1. Introduction

Child abuse is “any ill-treatment, attitude, or behavior that impacts a child’s physical, mental, or emotional growth and development”. This situation can harm the children’s sexual and social development and jeopardize their safety and health [1,2,3]. The poor socioeconomic status of families, population growth, rising unemployment rates, and immigration are some primary factors influencing children exposed to child abuse and neglect (CAN). Most child maltreatment cases occur at home, in schools, or on the streets, and the perpetrators are teachers, friends, other family members, neighbors, fathers, and mothers [4].

Children who have been exposed to traumatic experiences may develop a variety of mental health issues, including a propensity to engage in illicit trade or criminal behavior, as well as a predisposition to become addicted to drugs [2,4]. One of the negative consequences of CAN is that these children might abuse other children when they become adults in their immediate environment, creating a vicious cycle [5]. Teachers are the first professionals who have long-term, close relationships with children.5 As a result, teachers are in an excellent position to detect behavioral indicators. They can quickly identify unusual behaviors in children at various developmental stages. Children who have experienced abuse or neglect can have a reputation for being “bad kids” or being very challenging to manage or comprehend [6]. Reporting suspected child maltreatment is a professional and legally required duty for teachers. Additionally, teachers must be aware that any form of punishment, including time-outs, physical abuse, and removal from school or extracurricular activities, may have unintended negative consequences for maltreated or neglected children. The “National Child Abuse and Neglect Data System (NCANDS)” from the United States of America collects data to explain the prevalence of detecting and preventing “Child Abuse and Neglect” by focusing on significant child maltreatment concerns such as physical abuse, sexual abuse, neglect, and psychological maltreatment [7].

Child abuse is a problem that has always been considered unacceptable behavior in human societies, and it always affects more vulnerable children. Notwithstanding the endeavors of child support organizations to address this concern, child sexual harassment continues to be a worldwide dilemma. Even though societies have made scientific and cultural progress and parents and families are smarter than ever, the number of reported social harms to children keeps increasing. This has made many countries and international groups pay more attention to children and their problems and look for ways to solve them. Numerous educational tools such as lectures, brochures, posters, and mobile phone applications have educated the public [8,9,10].

Some researchers have recommended that there is a need to conduct more extensive testing of the effectiveness of CAN for educators to be better prepared to protect against the threat of CSN and sexual misconduct [11,12,13,14]. Chinese schoolchildren can learn about personal safety from the CSA preventive program. Other Chinese regions should strengthen and execute school-based CSA prevention education [14]. Some German findings imply that more targeted interventions to improve teachers’ decision making with probable child maltreatment scenarios, especially physical abuse, may help in recognizing and reporting maltreated children to child protection [12]. Nevertheless, each tool has its own set of constraints. Technology development has made it possible to emphasize using audiovisual aids and other teaching resources that appeal to both the senses of hearing and seeing [15]. The inclusion of authentic information in conjunction with audiovisual examples has been found to enhance participants’ comprehension and sense of readiness to handle specific situations [8]. The data from the Indian subcontinent were minimal regarding the awareness of CAN among the schoolteachers, which is considered to be very essential. Therefore, this study aimed to educate the teachers about CAN with the help of an audiovisual aid and assess their awareness regarding CAN through a pre-educational and post-educational intervention questionnaire.

## 2. Methodology

### 2.1. Ethical Approval

This cross-sectional study was conducted between 1 August 2020, and 1 December 2021 using 12-item questionnaires based on the Strengthening the Reports of Observational Studies in Epidemiology (STROBE) guidelines. The institutional ethics committee of Narayana Dental College and Hospital approved the study with IEC No. IEC/NDCH/2020/P-41. Teachers who were willing to participate in the study were included. A detailed patient information sheet about the nature and purpose of the study was also attached to the questionnaire.

### 2.2. Questionnaire Development and Validation

A pilot study was conducted among 30 schoolteachers from Nellore, Andhra Pradesh, India to estimate the sample size. Based on the prevalence of awareness obtained, the estimated sample size was 300. The questionnaire used in the study was developed by a standardized method under the following phases: (a) formation of a conceptual framework; (b) systematic development of questions; (c) refinement of the questions by focus group discussion; (d) pretesting; and (e) validity. The responses that were acquired from the pilot study were not incorporated into the final data analysis that was conducted. To ensure that the questionnaire is accurate, it was first validated, then translated into Telugu by a Telugu native speaker, and then modified before being distributed. English and Telugu versions of the translations were made available to the public. The individuals that took part made it straightforward to comprehend. A conceptual framework includes the key components that describe and define child abuse and neglect. After that, multiple strategies generated an initial pool of questions for each component. Questions were written as whole sentences, avoiding double negatives, two-edged questions, slang, and abbreviations. Later, questions in all the components were subjected to a thorough refinement process using focus group discussion between the researcher and participant. The researcher evaluated the questions regarding any confusion, deception, or unfamiliarity with the terms. The responses from the participants were analyzed, and necessary changes were made to the wording and terminology. The questionnaire was tested among 30 teachers to identify any difficulties in understanding the questionnaire and increase data accuracy. Data were collected from the participants through a face-to-face interview. The participants’ suggestions and opinions regarding understanding the wording and adequacy of questions were noted. After that, subject experts, including pediatric dentists and pediatricians, independently reviewed the questionnaire. The team verified whether the questions reflected their intended questions and evaluated the response options and their feasibility. Each expert rated each question as appropriate, inappropriate, or needing modification. Any remarks or recommendations for each item were also recorded.

### 2.3. Study Design, Setting, and Sample

This study is a prospective interventional study conducted with twenty-four government schools that have been selected from the complete list of government schools obtained from the district education officer of SPSR Nellore District, Andhra Pradesh, India. During the school visits, permission to conduct the study was provided by the headmaster, and teachers provided their informed consent to take part in the study. The questionnaire was distributed to all participant teachers. All the teachers were requested to fill out the forms, and all the filled forms were collected. Based on the previous study10, the sample size was determined to be 240.

### 2.4. Data Collection

A video recording has been created by the principal investigator, which explains the signs of “child abuse and neglect” and reports them to the higher authorities, child welfare, or any organization that protects the child from abuse and neglect. All the teachers from the respective schools have been shown the recorded video using audiovisual aids to deliver more knowledge about ‘child abuse and neglect’. After showing the recorded video to teachers, the same questionnaire form was provided to the teachers one more time to answer and collect information. The study was conducted in three phases, including 1st phase before the video, immediately after the video presentation (2nd phase), and 3rd phase after 90 days. The questionnaire was distributed to all the teachers, who were requested to fill out the forms, and all the filled-out forms were collected at the first visit. Later, a video recording created by the principal investigator, which explains CAN and the ways to report it to the higher authorities, child welfare, or any organization that protects the child from abuse and neglect, was shown to all the teachers. Immediately after the intervention, the same questionnaire was administered to assess the improvement in awareness. Finally, after three months, the awareness was reassessed using the same questionnaire as the first visit.

**Figure 1:**
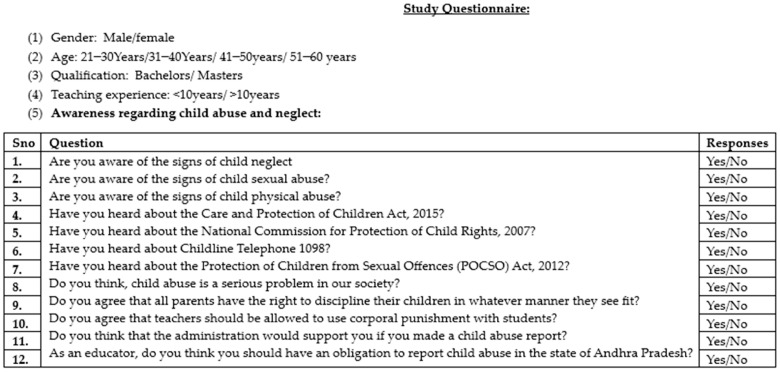
Questionnaire used in the study.

The questionnaire was divided into two sections; section one included gender, age, and experience, and section two involved 12 questions on awareness of CAN. Finally, the questionnaire consisting of 12 questions (Figure 1) regarding awareness was created by considering all the relevant changes the subject experts and the participants provided. The questionnaire involved two sections: demographic characteristics and awareness questionnaire. The demographic questions involved gender (male and female), age (>30 years, 31–40 years, 41–50 years, and 51–60 years), experience (<10 years and >10 years), and education (bachelor and master). The awareness questionnaire involved the Care and Protection of Children Act, 2015 [15], the National Commission for Protection of Child Rights, 2007 [16], and the Protection of Children from Sexual Offences (POCSO) Act, 2012 [17].

### 2.5. Statistical Analysis

The responses collected from all the participants were entered in the Microsoft Excel spreadsheet 2016. Fisher’s exact test was used for the data analysis to compare the responses after three visits. The comparisons were conducted based on age, gender, experience, and qualification. The multi-regression analysis was completed using. The Z Score Calculator for 2 Population Proportions was used to compare the percentages of results in various phases for all 12 questions. The statistical analysis was completed with the Windows version of the SPSS (version 17.0 software, Chicago, IL, USA). The level of significance was set at 0.05.

## 3. Results

Three-hundred-thirty teachers from 24 schools were recruited during the first visit. Among them, 30 participants were excluded as they did not appear during the three-month follow-up visit. Finally, 300 participants (108 males and 192 females) were included in the study. Moreover, 2.3% of the participants were between the ages of 21 to 30 years; 44.7% were between the ages of 31 to 40 years; 18.7% were between the ages of 41 to 50 years; and 34.3% were between the ages of 51 to 60 years. In the present study, 43% of the participants had less than ten years of experience, whereas 57% had greater than ten years of teaching experience. A statistically significant difference (*p* < 0.05) was observed among the males and females for the questions about the Care and Protection of Children Act and the POCSO Act during the baseline. Except for the questions about awareness of signs of sexual abuse in children, the National Commission for Protection of Child Rights, attitudes towards the seriousness of child abuse in our society, parents’ right to discipline their children, and corporal punishment by teachers immediately after the intervention at the second interval, and for the questions regarding awareness of the Care and Protection of Children Act, child line telephone, and POCSO Act at the three-month follow-up visit, all the other questions at all three intervals had no statistical significance (Table 1). Prior to the video presentation, the gender-based comparison was found to be statistically significant for Question 4 (Have you heard about the Care and Protection of Children Act, 2015?) and Question No. 7 (Have you heard about the Protection of Children from Sexual Offences (POCSO) Act, 2012?). Immediately after the presentation, the gender-based comparison showed statistical significance for Question 2 (Are you aware of the signs of child sexual abuse?), Question No. 8 (Do you think, child abuse is a serious problem in our society), Question 9 (Do you agree that all parents have the right to discipline their children in whatever manner they see fit?), and Question No. 10 (Do you agree that teachers should be allowed to use corporal punishment with students?). Nonetheless, after 30 days of the video presentation, the responses to Question 4 (Have you heard about the Care and Protection of Children Act, 2015?), Question No. 6 (Have you heard about Childline Telephone 1098?), and Question 7 (Have you heard about the Protection of Children from Sexual Offences (POCSO) Act, 2012?) showed significant differences for gender-based comparison.

The teachers’ awareness about child abuse and neglect was compared to the number of years of teaching experience. There was no statistically significant difference at any of the three points, except for the baseline questions about knowing about the National Commission for the Protection of Child Rights and the POSCO Act and immediately after the intervention regarding the question related to parents’ rights to discipline their children. After the three-month follow-up visit, for the questions regarding awareness of the Care and Protection of Children Act and the POSCO Act, all the participants in both the second and third intervals opted for “Yes” (Table 2). The experience-based comparison showed statistical significance for Questions No. 7 ((Have you heard about the Protection of Children from Sexual Offences (POCSO) Act, 2012?) and No. 12 (As an educator, do you think you should have an obligation to report child abuse in the state of Andhra Pradesh) prior to the video demonstration. Immediately after the presentation, there was no question about the experience-based correlation of a statistically significant difference. In the third phase, only Question No. 7 ((Have you heard about the Protection of Children from Sexual Offences (POCSO) Act, 2012?)) showed statistical significance for experience-based comparison.

The comparison of teachers’ responses regarding awareness of child abuse and neglect in all three phases for all 12 questions showed statistically significant differences except Question No. 8 (Do you think, child abuse is a serious problem in our society?). The responses for all the questions from the first phase to the second phase have been increased and also in the third phase. The details of the comparison of all three phases were summarized in Table 3. For Question No. 12 (As an educator, do you think you should have an obligation to report child abuse in the state of Andhra Pradesh), a high number of positive responses were observed in the first phase compared to the second and third phases. Similarly, for Question No. 11 (Do you think that the administration would support you if you made a child abuse report?), the teachers had a positive response in the second phase compared to the first and third phases. The comparison of responses for Questions 11 and 12 was statistically significant (*p* < 0.05).

The comparison among the three phases is described in Table 4. The comparison of phases 1 and 2 (Question No. 11) showed statistically no significant difference (*p* < 0.05), while the comparison of the second phase and third phase only (Question No. 9) showed statistically significant difference (*p* < 0.05). Meanwhile, the comparison for the first phase and third phase should be statistically significant (*p* < 0.05) for Questions 1, 2, 3, 4, 5, 6, 7, 10, and 11, whereas the responses for Questions 8, 9, and 12 showed statistically nonsignificant differences (*p* > 0.05).

## 4. Discussion

Addressing CAN requires the cooperation of the entire community, including social workers, teachers, other school employees, childcare providers, physicians and other healthcare workers, mental health professionals, and law enforcement officers [11]. Teachers can observe changes in children’s appearance and behavior because schools are one of the few places where children are observed virtually daily. Therefore, the current cross-sectional study was carried out to assess teachers’ awareness of CAN using pre- and post-educational intervention (audiovisual aid) questionnaires. They are particularly effective for measuring subject behavior, preferences, intentions, attitudes, and opinions [18]. In a study by Feng, teachers’ attitudes and perceived behavioral control about reporting were more significant than society’s expectations to report child abuse [19,20,21].

An audiovisual aid was used as an educational intervention in this study because it was thought to be the most effective way to teach and help people remember what they had learned. When factual information is provided along with audiovisual examples, the participant better understands the situation and feels more prepared to handle it. The AV aid provides a self-directed, comprehensive, evidence-based strategy for identifying and reporting CAN [22]. The audiovisual aid used in the current study was newly developed by the principal investigator in the native language, which may increase its benefits. It is distinctive in its pictorial approach and records basic, clear verbal instructions to keep the viewer’s attention.

In comparing awareness among the participants before and after the intervention, teachers have reported that a lack of awareness about child protection procedures is a deterrent to reporting [23]. Teachers’ responses favor attitude-based questions on particular children’s rights. Regarding considering CAN as a severe problem, there has been a unanimously positive attitude among teachers. However, most teachers showed negative attitudes, agreeing that parents have the right to discipline their children however they see fit. Most participants agreed that their administration would support them and that they should be obligated to report CAN. Even after the educational intervention, the people in the study could not remember what parts of the Indian Constitution protect their rights. This might be because this is an entirely new entity for this group. When comparing the awareness among teachers before and immediately after the educational intervention, their awareness changed regarding the CAN. Studies worldwide have revealed that teachers lack the awareness and self-assurance necessary to identify CAN correctly [24,25]. Gun et al. [26] stated that the teachers knew more about CAN and reporting after the training. This change was determined to be the result of the training. Likewise, in our study, AV aid helped improve teachers’ awareness regarding CAN.

A major change in the attitude of teachers was noticed after the educational intervention. The participants in our study agreed that parents and teachers should be allowed to use corporal punishment on children. This result is not entirely unexpected given that many parents and educators consider corporal punishment to be an appropriate form of punishment for children [27]. When comparing the attitude of the teachers immediately after the intervention with that of three months after the intervention, there was no change for most of the questions. Nevertheless, there was a variation in answering questions about parents’ rights to discipline their children. When comparing the pre- and post-education periods, the level of awareness of CAN improved due to education on the topic. Similar results were also reported in prior studies [28,29,30]. During the three visits, females showed higher awareness of a few questions. According to Demir’s survey of doctors, female doctors had more awareness about CAN than male doctors [31].

Regardless of the level of experience, almost all the teachers knew the same things about CAN during all three visits. However, teachers with one to ten years of experience knew more about the National Commission for Protection of Child Rights and the POSCO Act than teachers with more experience. Teachers with more experience seem likelier to report suspected CAN than teachers with low experience [24,32,33]. When comparing the attitudes of teachers based on their experiences during the first, second, and third intervals, all the study participants showed similar attitudes toward CAN [34,35,36]. The limitations of the present study are due to the highly homogeneous community of origin. Instead of including urban and rural regions, the sample only included teachers from government schools in the urban zone. Furthermore, only state-owned schools were included in the current study. Due to distinct employment requirements, government-run schoolteachers could be different from publicly run schoolteachers. Based on the findings of a recent systematic study, it has been established that the mistreatment of children might have negative consequences for the children, which may hinder their overall development [37,38,39,40,41]. The scenario will be completely different in European countries, Middle Eastern countries, North American countries, and the Indian subcontinent [37,38,39,40,41,42,43]. A Chinese group also highlighted the need to focus resources on child safety and improve national knowledge of child abuse in China based on a systematic review [44]. A recent Turkish study [43] reported that, in sum, teachers’ knowledge levels concerning child neglect and abuse significantly increased after training. Therefore, the Turkish national education system should develop and implement an intervention program to assist teachers in identifying incidents of child neglect and abuse. The findings were in agreement with the previous study. Collecting data from various contexts, including other provinces, private school establishments, and elementary and secondary schoolteachers, would be beneficial in arriving at better results [45,46,47,48,49]. Most recently Valtolina et al. [50] established a new technique for professionals that can help in establishing the early assessment of child neglect signs.

Additionally, the current study only evaluated self-reported data. A second data source, such as official documents, was not included. The study sample has been derived based on power analysis; nonetheless, to establish the conclusion with generalizability, it warrants further study with a large sample. The results were confined only to teachers from Andhra Pradesh, a southern state of India. Probably, this is a limitation of the study. The study only focused on awareness of CAN. The present study provides descriptive and qualitative findings. It also only included schoolteachers from 24 primary schools in the southern part of India. Additional research with a larger sample of Indian elementary schoolteachers from various states is needed to make the findings more applicable to a broader context. Additionally, using the data from the study on the information needs of teachers about CAN prevention, it is recommended to create a school-based CAN prevention model. This model needs to include the most up-to-date strategies for prevention, like making use of social media, which is a popular source of information for people of all ages. The practice of CAN was not evaluated, which could also be a potential limitation of the study. As a result, it has many shortcomings due to self-reported data, including biases in memory and social perception. Nevertheless, the results of this study indicate that a critical examination of the dynamics and connections between the child, the mandated reporters, the institutional system, the community, and society is essential. Nevertheless, there is a need for a standardized and comprehensive procedure.

## 5. Conclusions

According to the results of the current study, there needs to be more awareness about CAN among schoolteachers. Nevertheless, after education through AV aids, the awareness of CAN has improved considerably. It is anticipated that widespread use of this AV tool will enhance awareness of CAN and the reporting process. Early identification and reporting of CAN enable the implementation of appropriate follow-up actions to save children from further, possibly severe, harm.

## Figures and Tables

**Table 1:** Gender-based comparison of awareness at all three phases.

Questions/ Responses		1st Phase			2nd Phase			3rd Phase		
		Gender (%)		*p*-Value	Gender (%)		*p*-Value	Gender (%)		*p*-Value
		Male	Female		Male	Female		Male	Female	
Q1	Yes (%)	92.6	84.5	0.57	100	100		100	100	0.05
	No (%)	7.4	15.5		0	0		0	0	
Q2	Yes (%)	81.9	82.5	0.898	87%	100	<0.001 *	97.70	92.80	0.052
	No (%)	18.1	17.5		13%	0%		2.30	7.20	
Q3	Yes (%)	66	74.3	0.898	87	90.6	0.33	94.70	91.60	0.29
	No (%)	34	25.7		13	9.4		5.30	8.40	
Q4	Yes (%)	66	76.2	0.01 *	85.2	85.9	0.86	71.40	87.40	0.001 *
	No (%)	34	23.8		14.8	14.1		28.60	12.60	
Q5	Yes (%)	80.9	84	0.06	85.2	95.3	0.002 *	85.70	92.20	0.07
	No (%)	19.1	16		14.8	4.7		14.30	7.80	
Q6	Yes (%)	77.70%	78.2	0.50	91.7	90.6	0.762	96.20	89.80	0.03 *
	No (%)	22.3	21.8		8.3	9.4		3.80	10.20	
Q7	Yes (%)	66	81.6	0.003 *	85.2	90.6	0.154	95.50	87.40	0.015 *
	No (%)	34	18.4		14.8	9.4		4.50	12.60	
Q8	Yes (%)	95.5	94	0.57	93.5	100	<0.001 *	95.4	97.4	0.35
	No (%)	4.5	6		6.5	0		4.6	2.6	
Q9	Yes (%)	75.2	77.2	0.67	46.3	70.8	<0.001 *	75.9	78.1	0.66
	No (%)	24.8	22.8		53.7	29.2		24.1	21.9	
Q10	Yes (%)	27.8	29.3	0.77	30.6	17.7	0.01 *	20.4	18.8	0.73
	No (%)	72.2	70.7		69.4%	82.3		79.6	81.2	
Q11	Yes (%)	67.7	66.5	0.82	78.7	81.2	0.59	75.9	81.2	0.27
	No (%)	32.3	33.5		21.3%	18.8		24.1	18.8	
Q12	Yes (%)	95.5	94.	0.57	91.7	85.9	0.14	93.5	91.1	0.467
	No (%)	4.5	6		8.3	14.1		6.5	8.9	

* Significant; NS—nonsignificant.

**Table 2:** Comparison of awareness with the level of experience among the teachers at various intervals.

Questions/Responses		1st Phase			2nd Phase			3rd Phase		
		<10 Years	>10 Years	*p*-Value	<10 Years	>10 Years	*p*-Value	<10 Years	>10 Years	*p*-Value
Q1	Yes (%)	82.9	90.1	0.08	100.0	100.0	NA	100.0	100.0	NA
	No (%)	17.1	9.9		0	0		0	0	
Q2	Yes (%)	82.2	82.5	0.88	96.1	94.7	0.57	96.1	94.2	0.44
	No (%)	17.8	17.5		3.9	5.3		3.9	5.8	
Q3	Yes (%)	69.8	73.1	0.88	90.7	88.3	0.57	93.0	93.0	0.99
	No (%)	30.2	26.9		9.3	11.7		7.0	7.0	
Q4	Yes (%)	73.6	72.5	0.88	85.3	86.0	0.865	83.7	77.8	0.2
	No (%)	26.4	27.5		14.7	14.0		16.3	22.2	
Q5	Yes (%)	88.4	78.9	0.88	91.5	91.8	0.916	88.4	90.1	0.639
	No (%)	11.6	21.1		8.5	8.2		11.6	9.9	
Q6	Yes (%)	77.5	78.4	0.88	91.5	90.6	0.804	94.6	91.2	0.27
	No (%)	22.5	21.6		8.5	9.4		5.4	8.8	
Q7	Yes (%)	83.7	71.3	0.01 *	88.4	88.9	0.889	95.3	87.7	0.022 *
	No (%)	16.3	28.7		11.6	11.1		4.7	12.3	
Q8	Yes (%)	97.7	92.4	0.07	98.4	97.1	0.43	95.3	97.7	0.269
	No (%)	2.3	7.6		1.6	2.9		4.7	2.3	
Q9	Yes (%)	97.7	92.4	0.89	98.4	97.1	0.09	79.1	76.0	0.53
	No (%)	2.3	7.6		1.6	2.9		20.9	24.0	
Q10	Yes (%)	27.1	29.8	0.699	20.9	23.4	0.61	20.9	18.1	0.54
	No (%)	72.9	70.2		79.1	76.6		79.1	81.9	
Q11	Yes (%)	64.3	69.0	0.45	80.6	80.1	0.91	81.4	77.8	0.44
	No (%)	35.7	31.0		19.4	19.9		18.6	22.2	
Q12	Yes(%)	90.7	97.7	0.008 *	90.7	86.0	0.21	93.0	91.2	0.57
	NO (%)	9.3	2.3		9.3	14.0		7.0	8.8	

* Significant; NS—nonsignificant; NA-Not applicable.

**Table 3:** The comparison of teachers’ responses regarding awareness of child abuse and neglect in all three phases.

Questions/Responses		1st Phase	2nd Phase	3rdPhase	*p* Value
Q1	Yes	87.0	100.0	100.0	<0.001 *
	No	13.0	0.0	0.0	
Q2	Yes	82.3	95.3	95.0	<0.001 *
	No	17.7	4.7	5.0	
Q3	Yes	71.7	89.3	93.0	<0.001 *
	No	28.3	10.7	7.0	
Q4	Yes	73.0	85.7	80.3	0.001 *
	No	27.0	14.3	19.7	
Q5	Yes	83.0	91.7	89.3	0.003 *
	No	17.0	8.3	10.7	
Q6	Yes	78.0	91.0	92.7	<0.001 *
	No	22.0	9.0	7.3	
Q7	Yes	76.7	88.7	91.0	<0.001 *
	No	23.3	11.3	9.0	
Q8	Yes	94.7	97.7	96.7	0.128
	No	5.3	2.3	3.3	
Q9	Yes	76.3	62.0	77.3	<0.001 *
	No	23.7	38.0	22.7	
Q10	Yes	28.7	22.3	19.3	<0.001 *
	No	71.3	77.7	80.7	
Q11	Yes	67.0	80.3	79.3	<0.001 *
	No	33.0	19.7	20.7	
Q12	Yes	94.7	88.0	92.0	0.02 *
	No	5.3	12.0	8.0	

* Significant with *p* value less than 0.05.

**Table 4:** Intra-group comparison of awareness among three phases.

Questions	1st vs. 2nd Phases	2nd vs. 3rd Phases	1st and 3rd Phases
Q1	<0.001 *	1.00	<0.001 *
Q2	<0.001 *	0.849	<0.001 *
Q3	<0.001 *	0.114	<0.001 *
Q4	<0.001 *	0.082	0.034 *
Q5	0.001 *	0.33	0.024 *
Q6	<0.001 *	0.46	<0.001 *
Q7	<0.001 *	0.34	<0.001 *
Q8	0.056	0.46	0.24
Q9	<0.001 *	<0.001 *	0.77
Q10	0.075	0.37	0.007 *
Q11	<0.001 *	0.76	0.006 *
Q12	0.003 *	0.102	0.192

* Significant with *p* value less than 0.05.

## Data Availability

The data will be available upon request to the corresponding author.
